# Nuclear magnetic resonance spectroscopy of rechargeable pouch cell batteries: beating the skin depth by excitation and detection via the casing

**DOI:** 10.1038/s41598-020-70505-0

**Published:** 2020-08-13

**Authors:** Stefan Benders, Mohaddese Mohammadi, Christopher A. Klug, Alexej Jerschow

**Affiliations:** 1 Department of Chemistry, New York University, New York, USA; 2 United States Naval Research Laboratory, Washington, DC USA

**Keywords:** NMR spectroscopy, NMR spectroscopy, Batteries

## Abstract

Rechargeable batteries are notoriously difficult to examine nondestructively, and the obscurity of many failure modes provides a strong motivation for developing efficient and detailed diagnostic techniques that can provide information during realistic operating conditions. In-situ NMR spectroscopy has become a powerful technique for the study of electrochemical processes, but has mostly been limited to laboratory cells. One significant challenge to applying this method to commercial cells has been that the radiofrequency, required for NMR excitation and detection, cannot easily penetrate the battery casing due to the skin depth. This complication has limited such studies to special research cell designs or to ‘inside-out’ measurement approaches. This article demonstrates that it is possible to use the battery cell as a resonator in a tuned circuit, thereby allowing signals to be excited inside the cell, and for them to subsequently be detected via the resonant circuit. Employing this approach, ^7^Li NMR signals from the electrolyte, as well as from intercalated and plated metallic lithium in a multilayer (rolled) commercial pouch cell battery were obtained. Therefore, it is anticipated that critical nondestructive device characterization can be performed with this technique in realistic and even commercial cell designs.

## Introduction

Nuclear Magnetic Resonance (NMR) spectroscopy has proven to be a powerful technique for analyzing the properties of a broad array of materials under a wide range of physical conditions. Using radio frequency (rf) excitation, NMR signals from solids, liquids, and even gases can be obtained^1–3^. In situ NMR spectroscopy of electrochemical cells has become a highly active research area^4–6^. The most common experimental implementation involves placing the sample inside an inductor, typically a solenoid or a saddle coil, and adjusting the resonance of a tuned rf circuit with capacitors, inductors, and in some cases transmission lines. Using this approach, one can both deliver the rf excitation via an oscillating magnetic field and, by reciprocity, detect the NMR signal response produced. Unfortunately, for many real-world applications, e.g. commercial batteries, the material of interest is confined within a conductive container or casing, which effectively shields the sample from the rf excitation at all but the very low rf frequencies. One approach to bypass this problem, and to provide crucial device diagnostics, has been through the recently introduced inside-out MRI (ioMRI) technique, whereby one obtains information from the inside of the cell without needing the rf to penetrate into that volume^6–10^. While this method has become quite successful in terms of assessing the state of charge distribution and characterizing electrical current flow, it cannot currently distinguish directly between chemical species inside the cell since it does not provide spectroscopic information.

Previously, in another approach where the battery is part of a resonant circuit it has been demonstrated that some signals of interest could be obtained via a toroid cavity NMR resonator where a metal rod functions simultaneously as the working electrode of a compression coin cell and the central conductor of the toroid cavity^11,12^. In that work, a specially designed container was used, not an actual commercial-type cell design, which would contain a number of additional problematic components (in the case of a coin cell, that would include for example a stainless steel spring).

While a lot of basic battery materials research is being performed using coin cells, most battery chemistries change after initial upscaling from a coin cell design to a bigger pouch cell design, and this is the stage at which many new designs fail. It is therefore of prime interest to be able to study these more commercially relevant designs of rolled or stacked pouch cells at advanced stages of battery research or even for quality control of manufactured or deployed cells. Multilayer and rolled pouch cells, however, represent additional significant challenges for direct NMR investigation, mostly due to rf blockage by the conductors. This work demonstrates that by incorporating a pouch cell battery directly into a tuned rf circuit, and by adjusting the tuning conditions such that the signal is transmitted via the cell’s casing, it is possible to excite and detect NMR signals from the components inside the battery.

Notably, ^7^Li NMR spectra containing signals from key environments in the cell are presented. In particular, the ionic form associated with the electrolyte, the intercalated form in the graphite anode environment, as well as the metallic form due to built-up microstructure upon plating are clearly observed. Tracking these components hence becomes possible in a nondestructive fashion, thereby unlocking new characterization opportunities for crucial device diagnostics.

## Results and discussion

A pouch cell is typically made of a stack (or a roll) of closely spaced electrode layers with an electrolyte-soaked separator (e.g. based on glass fiber or polymer) in between. All layer thicknesses are typically of the order of 10–100 μm. The whole assembly is usually encased by a polymer-coated Al foil pouch (Figure S1).

It is not obvious how one could inject rf fields into such an object. For example, one could consider a cell a resonant cavity, that is, a body that can sustain a certain type of radiation based on its dimensions and the conductive wall boundary conditions. In this case, such an analysis would be misleading, because it would indicate that the only modes that can operate within the volume would have an extremely high frequency (based on the cell thickness of ~ 5 mm, this would be approximately 30 GHz, which would be far too large to be practical). Such considerations, however, are only valid in the cases where the cell consists of homogeneous conductor-free space.

**Figure 1:**
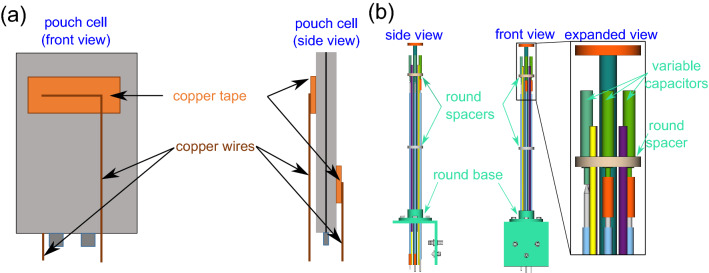
Schematic of pouch cell battery (grey) **(a)** with pads attached for feeding the rf into the cell via capacitive coupling. **(b)** Schematic of the home-built probe for this setup.

By making an electrical or capacitive connection via the pads shown in Fig. 1 on either side of the pouch, the two halves of the cell casing can be driven with a phase shift such as to create constructive interference of the waves within the volume. The presence of conductors inside the volume leads to more degrees of freedom for this model, so that additional modes can propagate within this volume far below the cutoff frequency^13^. Inspiration can also be drawn from the field of wireless power delivery where both inductive and capacitive approaches have been used to deliver energy across a variety of barriers, although generally at much lower frequencies than those used in this work^14,15^.

**Figure 2:**
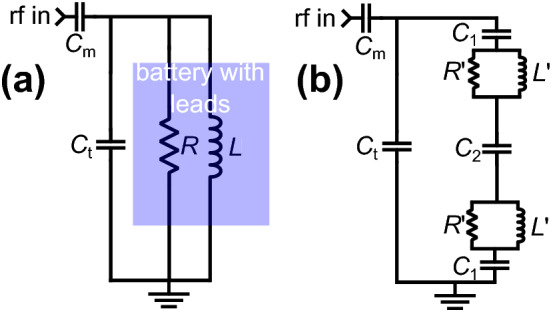
Circuit diagrams relevant to the NMR probes used in this work: **(a)** standard series-matched parallel-tuned resonant circuit used in many NMR probes—the blue box highlights the inductor and effective parallel resistor, those components which are effectively replaced by the battery. **(b)** The circuit diagram used in the modeling of the final rf circuit where ${C}_{2}$ represents the capacitance between the two aluminum sides of the pouch cell, each of which has an effective $R{^{\prime}}$ and $L{^{\prime}}$. ${C}_{1}$ represents the capacitance between the copper tape and one side of the aluminum pouch.

Based on these considerations, suitable tuning conditions for a pouch cell in order to transmit rf at the ^7^Li resonance frequency and detect the signal response are identified. NMR probes are typically tuned to the frequency of interest by either series or parallel tuning and matching circuits. Such circuits transform the impedance of the resonant circuit to a specific real resistance (typically 50 $\Omega$) for optimal power transmission through a similarly matched transmission line. A generic series-matched parallel-tuned resonant circuit^16^ is shown in Fig. 2a. The inductor *L* arises from the rf coil, and the resistor *R* is the effective Ohmic parallel resistance. Note that *R* arises originally from the small resistance of the wires of the circuit and the inductor (typically a fraction of 1 $\Omega$), and the fairly large resulting effective parallel resistance *R* (~ 50–100 k$\Omega)$ is a consequence of the transformation by the inductance and capacitance. The unloaded quality factor of this circuit is defined as *Q*_0_ ≡ *R*⁄ω_0_*L*, related to the circuit’s recovery time and observed signal size. For the ^7^Li resonance frequency of interest, 155 MHz, in our case (at a magnetic field of *B*_0_ = 9.4 T), typical values for a tuned circuit with standard rf coils could be *L* = 0.4 μH and *R* = 50 k$\Omega$. This circuit can be matched to 50 $\Omega$ by the use of matching capacitance *C*_m_ and tuning capacitance *C*_t_, of 0.65 pF and 1.99 pF, respectively, yielding a quality factor of ${Q}_{0}=$ 128.4. Additional tuning and matching combinations for the simple resonant circuit of Fig. 2a are shown in Table S1.

As a next step, the strategy for tuning and matching a battery cell that is connected is examined as shown in Fig. 1. In a first approximation, one could consider the cell as represented by the lumped circuit elements as shown in Fig. 2b. Such lumped circuit models are often used in electrical impedance spectroscopy^17–20^ to describe how different device components contribute to the overall impedance at a given frequency. These models vary greatly, depending on the frequency ranges examined, but also depending on the level of detail that one wishes to describe with this approach. For this purpose, a ‘coupling’ capacitance *C*_1_ that describes the overall capacitance at each pad due to the connection made between the copper tape and the casing material as well as the inner cell compartment is included. Next, a parallel arrangement of some resistance *R*′ and inductance *L*′, to reflect the influence of electrodes, as well as current migration through the electrolyte is incorporated. Note that for the cell under investigation, and for typical pouch cells, one side of the casing is not in full electrical contact with the other due to the nature of the material (polymer-coated Al foil). The capacitance between the two halves is included as the series capacitance C_2_, which also describes the overall effect of several stacked electrode layers.

**Figure 3:**
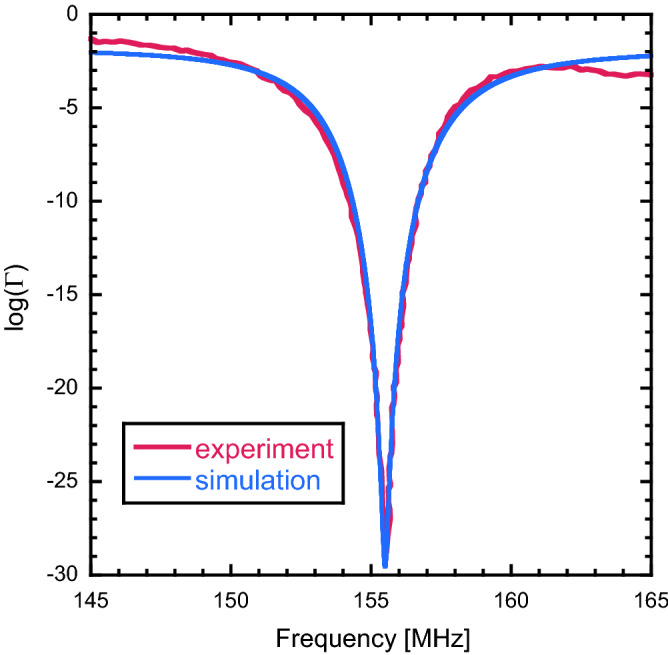
Plot of experimental (red) tuning curve obtained for our battery-as-coil circuit along with a tuning curve calculated using the model circuit of Fig. 2b (blue) and the equations described in the Supporting Information. The loaded *Q*, as determined by the width of the tuning minimum was 29. The parameters used in the calculation were: *L*′ = 0.42 μH, *R*′ = 25 k$\Omega$, *C*_1_ = *C*_2_ = 40 pF, *C*_m_ = 0.74 pF, *C*_t_ = 0.63 pF.

Turning to the resonant circuit shown in Fig. 2b, similar impedance calculations as for the circuit in Fig. 2a to estimate the values for ${C}_{1}$, ${C}_{2}$, $R{^{\prime}}$ and ${L}^{^{\prime}}$ can be employed. Experimentally, it was found that resonant and matching conditions could be reached using ${C}_{m}$ and ${C}_{t}$ within a range of 0.5 to 4 pF while the loaded *Q* was in the range of 20–100. With these experimental values, the remaining parameters can be determined. The Supporting Information lists additional analyses allowing the determination of the optimal tuning/matching conditions and to narrow down the range of the parameters. These analyses show, that the circuits in Fig. 2 are equivalent for very large ${C}_{1}$ and ${C}_{2}$, with ${R}^{^{\prime}}=R/2$ and ${L}^{^{\prime}}=L/2$ and ${C}_{1}={C}_{2}=5000$ (Tables S1 and S2). Given the typical tuning curve shown in Fig. 3 which yields a loaded $Q$ of 29, a curve can be simulated with *L*′ =  0.42 μH, *R*′ = 25 k$\Omega$, *C*_1_ = *C*_2_ =  40 pF, *C*_m_ =  0.74 pF, *C*_t_ = 0.63 pF which matches the experimental tuning curve well . ${C}_{m}$ and ${C}_{t}$ resulting from calculations such as those summarized in Table S2 suggest that ${C}_{1}$ and ${C}_{2}$ are on the order of 10 pF or larger, slightly above our observations. Furthermore, an estimate of ${C}_{1}$ can be obtained by considering the area, $A$, of the copper tape (Fig. 2) as 2 cm^2^, and assuming the effective distance between the copper tape and the aluminum case to be $d$ = 0.1 mm, with the relative permittivity of the medium being 2–3 for the polymer film of the casing. This calculation yields 40–60 pF for ${C}_{1}$, which is similar to the value shown in Fig. 3. In summary, using the experimentally determined *C*_m_, *C*_t_, and *Q* and estimates of *C*_1_ and *C*_2_, the simple circuit model of Fig. 2b describes the tuning properties of our circuit and leads to tuning curves which match those experimentally measured.

**Figure 4:**
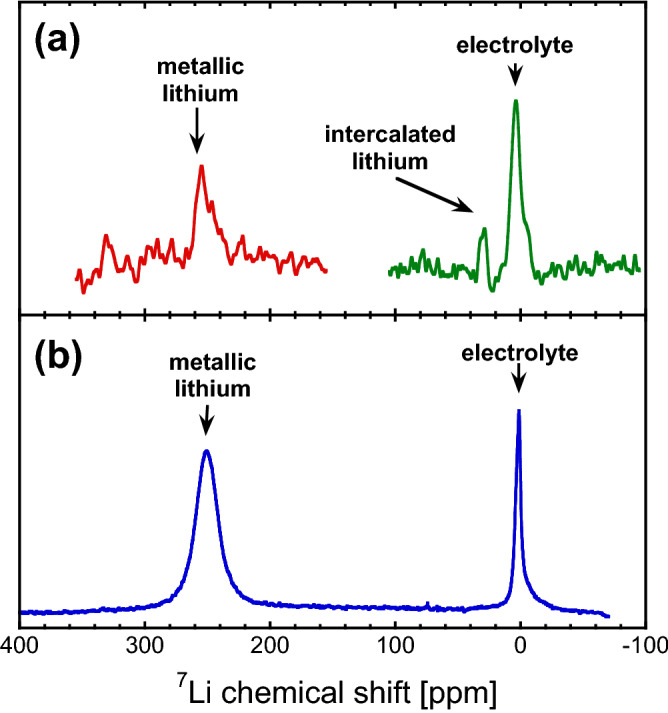
^7^Li NMR spectra obtained for the battery-as-coil setup using a Hahn echo preparation **(a)** and for a reference battery cell using a traditional solenoid coil and single pulse acquisition **(b)**. Due to limited excitation bandwidth, the spectra in **(a)** were obtained from two separate measurements, one where the spectrometer frequency was at 255 ppm (left) and one where the spectrometer frequency was 5 ppm (right).

Figure 4 shows the NMR spectra obtained from the cell with this setup. The spectra display clear evidence of the characteristic signals of electrolyte (ionic) lithium (near 0 ppm), metallic lithium (near 260 ppm), and lithium intercalated into graphite (near 30 ppm). Background ^7^Li signals in the probe can be neglected and therefore the observation of a signal at 155.5 MHz indicates that signal from within the pouch cell battery is obtained. (The nearest NMR resonance frequencies are ^31^P at 162.0 MHz, ^119^Sn at 149.2 MHz, ^117^Sn at 142.5 MHz.) Furthermore, signals from probe ringing can be ruled out because the spectra were acquired using Hahn echoes with sufficient echo times and phase cycles that would eliminate the signatures of ringing.

The assignment of the signals is further corroborated by comparing the spectra to those obtained using a solenoid coil with a reference lithium metal cell. A very good correspondence between the shifts observed for electrolyte ^7^Li, near 0 ppm, and metallic lithium, near 260 ppm, is found here. The intercalated lithium cannot be observed in this particular cell, but literature suggests a peak in this area^21,22^.

The detection of all these components is of great interest in battery research. The quantification and localization of electrolyte lithium is relevant for the study of electrolyte gradients, the assessment of electrolyte degradation, leakage, and proper distribution. The detection of intercalated lithium is relevant for the quantification of anodic energy storage. The quantification of metallic lithium is characteristic for the buildup of lithium microstructure, including lithium dendrites, which is often a degradative process in cells, and indicates the onset of failure modes^17^. It is interesting to observe that these metallic lithium signals could be detected in a commercial cell with a graphitic anode. In such cells metallic lithium would only ever occur in such a cell following a degradative process. For example, this process may be a consequence of overcharging or fast charging.

While DNP was recently demonstrated to provide an in-situ characterization capability^23^, we do not believe that this approach could be used here for signal enhancement, due to the difficulties of injecting microwaves into the conductively shielded assembly. Alternatively, one might envision enhancing sensitivity by employing larger magnetic fields, but this improvement would have to be balanced against the challenges of tuning to higher frequencies and stronger inhomogeneity effects.

The presented technique could further be combined with MRI and potentially site-resolved nutation experiments in order to obtain further insights into the distribution of RF fields within the cell, and provide more detailed device characterization and speciation.

## Methods

To incorporate the battery cell into the resonant circuit, a simple NMR probe was designed and constructed. It is compatible with a Bruker Ultrashield 9.4 T Avance I spectrometer containing a Bruker Micro2.5 gradient assembly with an inner diameter of 40 mm. The layout of the probe was optimized for future flexibility, e.g., the ability to incorporate up to four high-voltage variable capacitors for multiple-tuning and a large flexibility in sample geometry. Additionally, tubes were incorporated for frame cooling, electrical connections and a middle tube for additional accessory items. Every effort was made to use readily available parts, e.g., tubing with non-metric diameters. The drawings for the probe are shown in Fig. 1.

The NMR parameters used in these experiments are given in Table 1. For the spin echo experiments, a 16-step phase cycle was employed (ϕ_1_ = x, y, − x, − y, x, y, − x, − y, x, y, − x, − y, x, y, − x, − y; ϕ _2_ = x, x, x, x, y, y, y, y, − x, − x, − x, − x, − y, − y, − y, − y; ϕ _rec_ = x, − y, − x, y, − x, y, x, − y, x, − y, − x, y, − x, y, x, − y). It was difficult to obtain accurate estimates of the optimal pulse lengths in the spin echo experiments due to the large inhomogeneity of the internal rf fields. The pulse lengths used were chosen based on an estimation extracted from a series of single-pulse experiments (Figure S2).

**Table 1:** NMR parameters used.

Experiment	Recycle delay	Echo time	Pulse 1	Pulse 2	Number of averages	Transmitter frequency
Metal	0.4 s	1.1 ms	337 µs @ ~ 240 W	674 µs @ ~ 240 W	40,960	155.5488 MHz
Electrolyte	1/2/0.75 s	1.1 ms	337 µs @ ~ 240 W	674 µs @ ~ 240 W	32,768/32,768/81,920	155.5100 MHz
Reference	0.4 s	n.a	16 µs		1536	155.5482 MHz

Battery cells and description: The actual dimensions of the pouch cell battery used in this work were approximately 40 mm × 30 mm × 5 mm). The cell was a PowerStream (Utah, US) Jelly rolled lithium ion battery with 600 mAh capacity. The graphite and NMC electrodes are rolled in twelve active layers and packed inside an aluminum pouch case. The battery is made from graphite anode, aluminum and copper current collectors. The cathode is made of Co (44.76%), O (33.20%), Ni (4.79%), Mn (2.99%). It was cycled using a current of 300 mA (a charge/discharge rate of 0.5 C) several times before integrating it into the circuit.

Contacts between the pads and the cell were improved by using fine sandpaper to remove some of the polymer coating on each face of the pouch cell while avoiding puncturing the very thin aluminum metal casing. This step may not be needed since the resonating conditions are based on capacitive coupling.

## Conclusions

It was shown here that it is possible to allow rf irradiation to penetrate into the inside compartment of Li-ion battery cells, excite and detect NMR signals and record NMR spectra. The key to the success of this approach was the incorporation of the cell directly into the tuned rf circuit via capacitive coupling. Placing the capacitively coupled pads on either side of the cell allows driving the casing with a phase difference and thus to generate the requisite oscillating magnetic field inside. While in our initial experiment the absolution magnitude of these internal fields was small and there was evidence for significant inhomogeneity, it was possible to obtain a ^7^Li NMR spectrum of the three most important lithium environments in a cell: ^7^Li in the electrolyte, graphite-intercalated lithium, and metallic lithium. These three environments reflect critical device parameters, which could be monitored nondestructively over time and at different stages of a battery’s life cycle. Modifications in the coupling of the pads to the cell could lead to improvements in the internal fields. Future implementations could also be used to provide the possibility to spatially resolve species on the basis of this battery tuning approach for advanced device characterization.

## Supplementary information

Supplementary Information.
