# Supplementary material for: Nuclear magnetic resonance spectroscopy of rechargeable pouch cell batteries: beating the skin depth by excitation and detection via the casing
Source: Sci Rep. 2020 Aug 13;10:13781. doi: 10.1038/s41598-020-70505-0 (PMC7426951; doi:10.1038/s41598-020-70505-0)
Supplement: Supplementary file 1 — Supplementary Information. [file 41598_2020_70505_MOESM1_ESM.pdf]

Nuclear Magnetic Resonance Spectroscopy of Rechargeable Pouch Cell Batteries: Beating the Skin Depth by Excitation and Detection Via the Casing.

Stefan Benders[a], Mohaddese Mohammadi[a], Christopher A. Klug[a,b*], Alexej Jerschow[a*]

[a]Department of Chemistry, New York University, New York, United States
[b]United States Naval Research Laboratory, Washington, District of Columbia, United States

Supporting Information

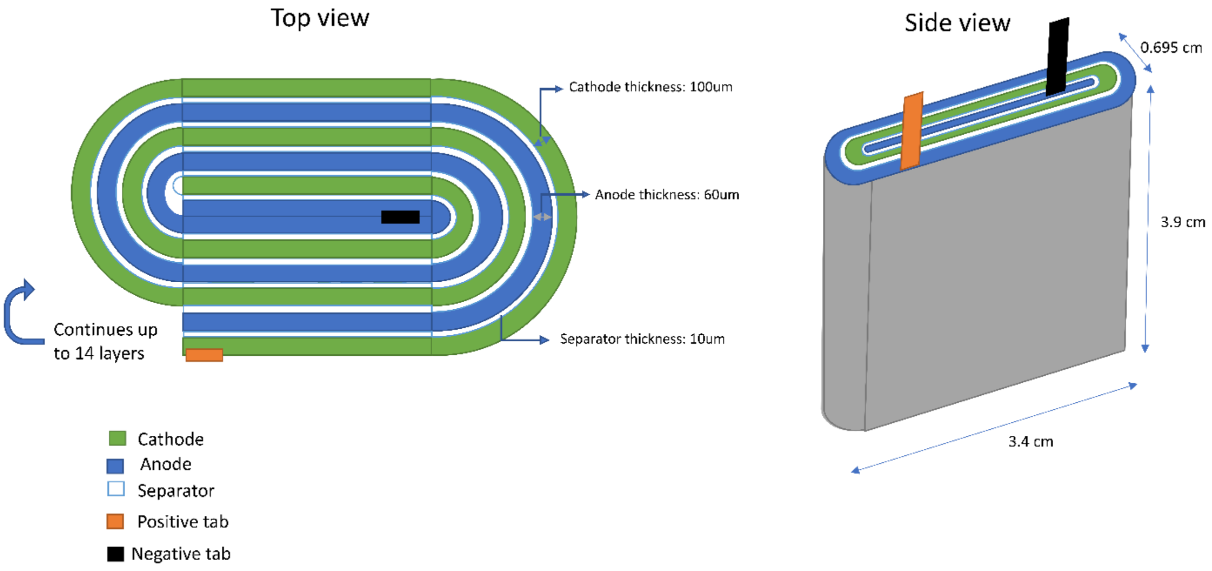

Figure S1: Schematic of a jelly-rolled pouch cell. A long sheet of anode, separator and cathode is rolled into shape. The anode is 60 μm thick, the separator 10 μm, and the cathode 100 μm. The dimensions of the cell are $34 \times 39 \times 6.95\ mm^3$.

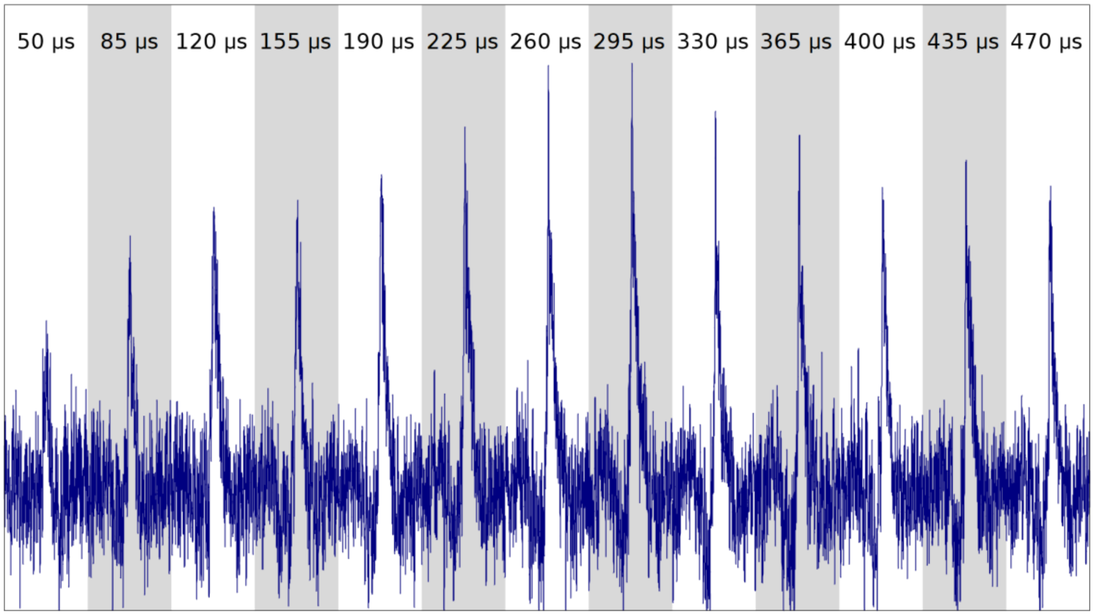

Figure S2: Nutation curve for the electrolyte peak at ~240 W pulse power. Parameters were 1024 scans, 100 kHz spectral width, a recycle delay of 5 s and a transmitter frequency of 155.510 MHz. The excitation pulse length was incremented in steps of 35 µs from 50 µs to 470 µs.

Here we derive the simple equations used to generate plots of the tuning and matching capacitance for the two circuits shown in Figure 2 as a function of the impedance of other circuit components. For the typical series-matched parallel-tuned circuit shown in Figure 2a we can write an expression for the impedance as:

$$Z_p = \frac{1}{Z_{C_m}} + \left(\frac{1}{Z_{C_t}} + \frac{1}{Z_L} + \frac{1}{R}\right)^{-1} = -\frac{j}{\omega C_m} + \left(j\omega C_t - \frac{j}{\omega L} + \frac{1}{R}\right)^{-1} \quad \text{(S.1)}$$

Tuning the circuit involves finding C_m and C_t so that Z_p=50 Ω, *i.e.*, $Re(Z_p) = R_0 = 50\ \Omega$ and $Im(Z_p) = 0\ \Omega$. Returning to (S.1)

$$Z_p = -\frac{j}{\omega C_m} + \left(\frac{1}{\frac{1}{R} + j\left(\omega C_t - \frac{1}{\omega L}\right)}\right) = -\frac{j}{\omega C_m} + \left(\frac{\frac{1}{R} - j\left(\omega C_t - \frac{1}{\omega L}\right)}{\frac{1}{R^2} + \left(\omega C_t - \frac{1}{\omega L}\right)^2}\right) \quad \text{(S.2)}$$

$$R_0 = \left(\frac{\frac{1}{R}}{\frac{1}{R^2} + \left(\omega C_t - \frac{1}{\omega L}\right)^2}\right) \text{ and } \frac{1}{\omega C_m} = -\left(\frac{\left(\omega C_t - \frac{1}{\omega L}\right)}{\frac{1}{R^2} + \left(\omega C_t - \frac{1}{\omega L}\right)^2}\right) \quad \text{(S.3 \& S.4)}$$

We can use (S.3) to solve for C_t

$$\left(\omega C_t - \frac{1}{\omega L}\right)^2 = \frac{1}{RR_0} - \frac{1}{R^2} = \frac{R-R_0}{R^2 R_0} \tag{S.5}$$

$$\left(\omega C_t - \frac{1}{\omega L}\right) = \pm\frac{1}{R}\sqrt{\frac{R}{R_0} - 1} \tag{S.6}$$

$$C_t = \frac{1}{\omega^2 L}\left(1 \pm \frac{\omega L}{R}\sqrt{\frac{R}{R_0} - 1}\right) \tag{S.7}$$

Once we have C_t, we can use (S.4) to obtain C_m

$$C_m = -\frac{1}{\omega}\left(\frac{\frac{1}{R^2} + \left(\omega C_t - \frac{1}{\omega L}\right)^2}{\left(\omega C_t - \frac{1}{\omega L}\right)}\right) \tag{S.8}$$

Note that one can use (S.5) and (S.6) to simplify (S.8)

$$C_m = -\frac{1}{\omega}\left(\frac{\frac{1}{RR_0}}{\pm\frac{1}{R}\sqrt{\frac{R}{R_0} - 1}}\right) \tag{S.9}$$

$$C_m = -\frac{1}{\omega}\left(\pm\sqrt{R_0(R - R_0)}\right)^{-1} \tag{S.10}$$

This agrees with reference [1].

We can apply a similar approach to the battery circuit shown in Figure 2b starting with the equation for the total impedance:

$$Z_b = Z_{C_m} + \left(\frac{1}{Z_{C_t}} + \frac{1}{2Z_{C_1} + Z_{C_2} + 2\left(\frac{1}{Z_{L'}} + \frac{1}{Z_{R'}}\right)^{-1}}\right)^{-1} \tag{S.11}$$

$$Z_b = -\frac{j}{\omega C_m} + \left(j\omega C_t + \frac{1}{-\frac{2j}{\omega C_1} - \frac{j}{\omega C_2} + 2\left(-\frac{j}{\omega L'} + \frac{1}{R'}\right)^{-1}}\right)^{-1} \tag{S.12}$$

$$Z_b = -\frac{j}{\omega C_m} + \left(j\omega C_t + \frac{1}{-\frac{2j}{\omega C_1} - \frac{j}{\omega C_2} + 2\left(\frac{\omega L' R'}{\omega L' - jR'}\right)}\right)^{-1} \tag{S.13}$$

$$Z_b = -\frac{j}{\omega C_m} + \left(j\omega C_t + \frac{1}{-\frac{2j}{\omega C_1} - \frac{j}{\omega C_2} + 2\left(\frac{\omega L' R'(\omega L' + jR')}{\omega^2 (L')^2 + (R')^2}\right)}\right)^{-1} \tag{S.14}$$

$$Z_b = -\frac{j}{\omega C_m} + \left(j\omega C_t + \frac{1}{\frac{2\omega^2 (L')^2 R'}{\omega^2 (L')^2 + (R')^2} + j\left(\frac{2\omega L' (R')^2}{\omega^2 (L')^2 + (R')^2} - \frac{2j}{\omega C_1} - \frac{j}{\omega C_2}\right)}\right)^{-1} \tag{S.15}$$

Note that in the limit $C_1, C_2 \rightarrow \infty$, (S.12) becomes

$$Z_b = -\frac{j}{\omega C_m} + \left(j\omega C_t - j\frac{1}{2\omega L\prime} + \frac{1}{2R\prime}\right)^{-1} \tag{S.16}$$

which is the same as (S.1) when $2L' = L$ and $2R' = R'$

Returning to (S.15) and using the substitutions:

$$A = \frac{2\omega^2 (L')^2 R\prime}{\omega^2 (L')^2 + (R')^2} \text{ and } B = \frac{2\omega L' (R')^2}{\omega^2 (L')^2 + (R')^2} - \frac{2j}{\omega C_1} - \frac{j}{\omega C_2} \qquad \text{(S.17) \& (S.18)}$$

we can rewrite (S.15) as

$$Z_b = -\frac{j}{\omega C_m} + \left(j\omega C_t + \frac{1}{A + jB}\right)^{-1} \tag{S.19}$$

$$Z_b = -\frac{j}{\omega C_m} + \left(j\omega C_t + \frac{A - jB}{A^2 + B^2}\right)^{-1} \tag{S.20}$$

$$Z_b = -\frac{j}{\omega C_m} + \left(\frac{A^2 + B^2}{j\omega C_t (A^2 + B^2) + A - jB}\right) \tag{S.21}$$

$$Z_b = -\frac{j}{\omega C_m} + \left(\frac{(A^2 + B^2)\left[A + j\left(B - \omega C_t (A^2 + B^2)\right)\right]}{\left(B - \omega C_t (A^2 + B^2)\right)^2 + A^2}\right) \tag{S.22}$$

Remembered that at tuning $Z_b = R_0$, we can now write

$$R_0 = \frac{(A^2 + B^2)A}{\left(B - \omega C_t (A^2 + B^2)\right)^2 + A^2} \tag{S.23}$$

We can now solve for C_t in steps

$$\left(B - \omega C_t (A^2 + B^2)\right)^2 = \frac{(A^2 + B^2)A}{R_0} - A^2 \tag{S.24}$$

$$B - \omega C_t (A^2 + B^2) = \pm\sqrt{\frac{(A^2 + B^2)A}{R_0} - A^2} \tag{S.25}$$

$$C_t = \frac{1}{\omega (A^2 + B^2)}\left[B \pm \sqrt{\frac{(A^2 + B^2)A}{R_0} - A^2}\right] \tag{S.26}$$

Using the fact that $Z_b$is pure real at tuning allows

$$\frac{1}{\omega C_m} = \frac{(A^2 + B^2)\left(B - \omega C_t (A^2 + B^2)\right)}{\left(B - \omega C_t (A^2 + B^2)\right)^2 + A^2} \tag{S.27}$$

$$C_m = \frac{1}{\omega}\left(\frac{\left(B-\omega C_t\left(A^2+B^2\right)\right)^2+A^2}{(A^2+B^2)\left(B-\omega C_t(A^2+B^2)\right)}\right) \quad \text{(S.28)}$$

Note that in the limit $C_1, C_2 \to \infty$

$$A = \frac{2\omega^2\left(L'\right)^2 R'}{\omega^2(L')^2+(R')^2} \text{ and } B = \frac{2\omega L'\left(R'\right)^2}{\omega^2(L')^2+(R')^2} = \left(\frac{R'}{\omega L'}\right)A \quad \text{(S.29) \& (S.30)}$$

$$A^2 + B^2 = \frac{\left(2\omega L'R'\right)^2\left(\omega^2\left(L'\right)^2+\left(R'\right)^2\right)}{(\omega^2(L')^2+(R')^2)^2} = \frac{\left(2\omega L'R'\right)^2}{\omega^2(L')^2+(R')^2} \quad \text{(S.31)}$$

$$R_0 = \frac{\left(A^2+B^2\right)A}{\left(B-\omega C_t(A^2+B^2)\right)^2+A^2} \quad \text{(S.32)}$$

$$R_0 = \frac{\left(A^2+B^2\right)A}{A^2+B^2-2\omega C_t B(A^2+B^2)+\left(\omega C_t(A^2+B^2)\right)^2} \quad \text{(S.33)}$$

$$R_0 = \frac{A}{1-2\omega C_t B+\omega^2 C_t^2(A^2+B^2)} \quad \text{(S.34)}$$

Focusing on the denominator:

$$1 - 2\omega C_t B + \omega^2 C_t^2(A^2 + B^2) = \frac{\omega^2\left(L'\right)^2+\left(R'\right)^2-2\omega C_t 2\omega L'\left(R'\right)^2+\omega^2 C_t^2\left(2\omega L'R'\right)^2}{\omega^2(L')^2+(R')^2} \quad \text{(S.35)}$$

$$1 - 2\omega C_t B + \omega^2 C_t^2(A^2 + B^2) = \frac{\omega^2\left(L'\right)^2+\left(2\omega^2 C_t L'R'-R'\right)^2}{\omega^2(L')^2+(R')^2} \quad \text{(S.36)}$$

$$1 - 2\omega C_t B + \omega^2 C_t^2(A^2 + B^2) = \frac{\omega^2\left(L'\right)^2+\left(2\omega L'R'\right)^2\left(\omega C_t-\frac{1}{2\omega L'}\right)^2}{\omega^2(L')^2+(R')^2} \quad \text{(S.37)}$$

Now back to R_0

$$R_0 = \frac{2\omega^2\left(L'\right)^2 R'}{\omega^2(L')^2+(2\omega L'R')^2\left(\omega C_t-\frac{1}{2\omega L'}\right)^2} \quad \text{(S.38)}$$

$$R_0 = \frac{2R'}{1+(2R')^2\left(\omega C_t-\frac{1}{2\omega L'}\right)^2} \quad \text{(S.39)}$$

$$R_0 = \frac{\frac{1}{2R'}}{\frac{1}{\left(2R'\right)^2}+\left(\omega C_t-\frac{1}{2\omega L'}\right)^2} \quad \text{(S.40)}$$

This is the same as (S.3) with $2R' = R$.

We can now use (S.7) and (S.8) for the normal circuit and (S.26) and (S.28) for the battery circuit to make plots shown below. By calculating the impedance as a function of frequency we are also able to determine the loaded Q for the circuits.

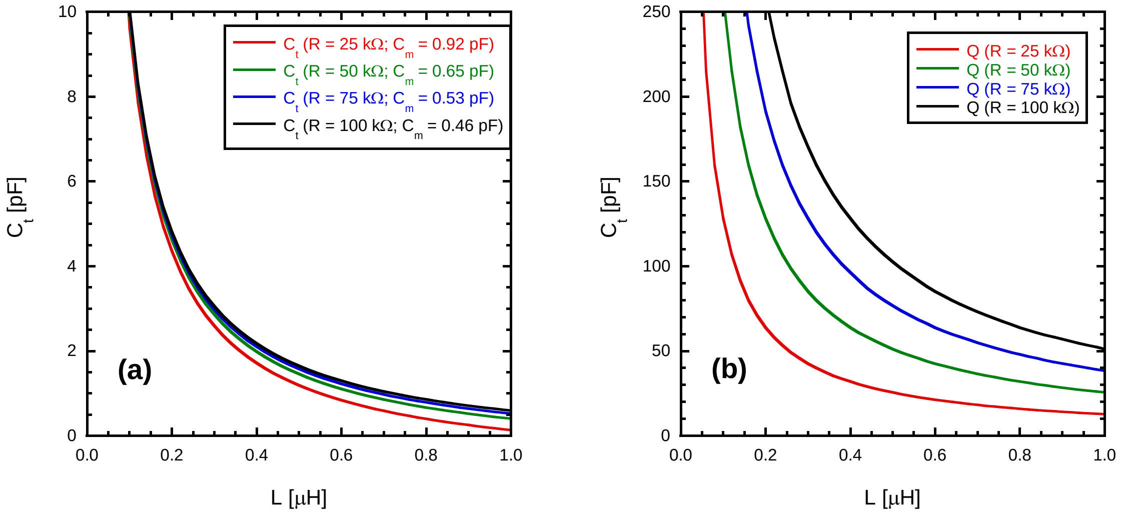

Figure S3: (a) Plot of the calculated optimal tuning capacitance, C_t, of the normal circuit as a function of the inductance, L, of the coil for a range of effective parallel resistances, R; Note that the matching capacitance is determined solely by R and R_0 (50 Ω)—see equation (S.10). (b) Plot of the loaded Q of the normal circuit as a function of the inductance, L, of the coil for a range of effective parallel resistances, R. The loaded Q was found from direct calculations of the impedance as a function of frequency and quantitatively agrees with $Q = Q_0/2 = R/(2\omega_0 L)$.

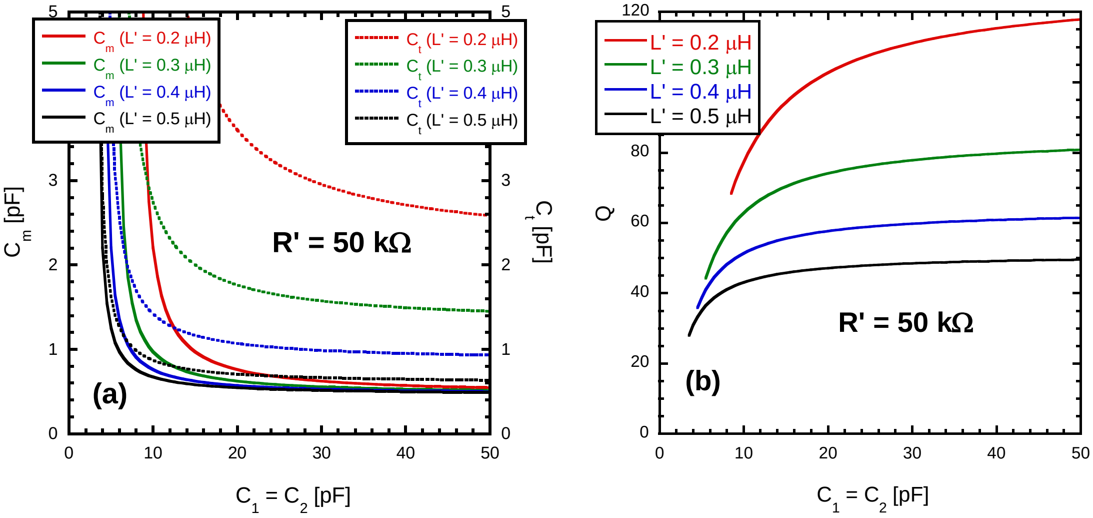

Figure S4: Consider the case $C_1 = C_2$: (a) Plot of the calculated optimal tuning capacitance, C_t, and matching capacitance, C_m, of the battery-as-coil circuit as a function of the two capacitances, C_1 and C_2, for a range of inductances, L', and an effective parallel resistance, R', of 50 kΩ; (b) Plot of the loaded Q of the circuit as a function of the two capacitances, C_1 and C_2. The loaded Q was found from direct calculations of the impedance as a function of frequency.

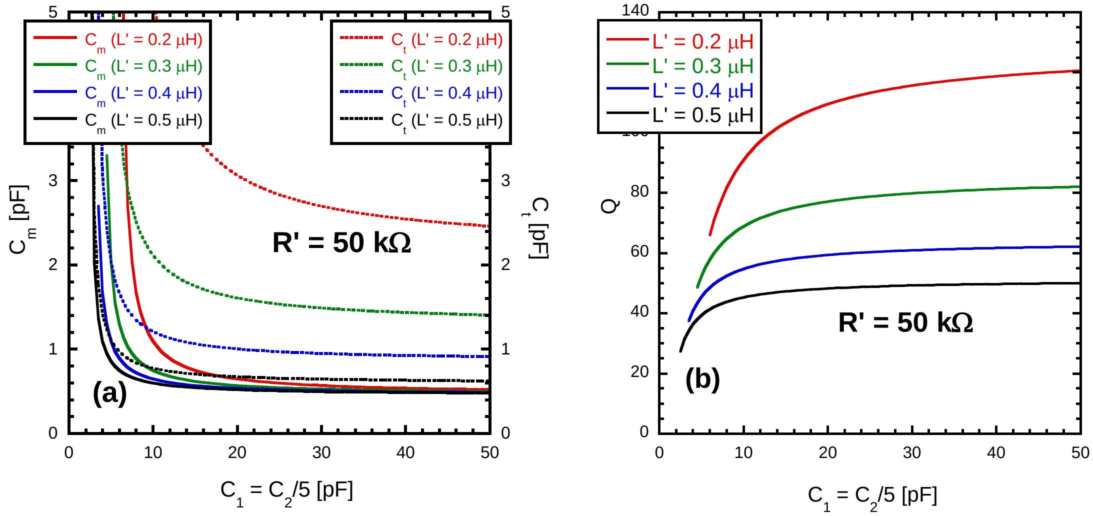

Figure S5: Consider the case $C_1 = C_2/5$: (a) Plot of the calculated optimal tuning capacitance, C_t, and matching capacitance, C_m, of the battery-as-coil circuit as a function of the two capacitances, C_1 and C_2, for a range of inductances, L', and an effective parallel resistance, R', of 50 kΩ; (b) Plot of the loaded Q of the circuit as a function of the two capacitances, C_1 and C_2. The loaded Q was found from direct calculations of the impedance as a function of frequency.

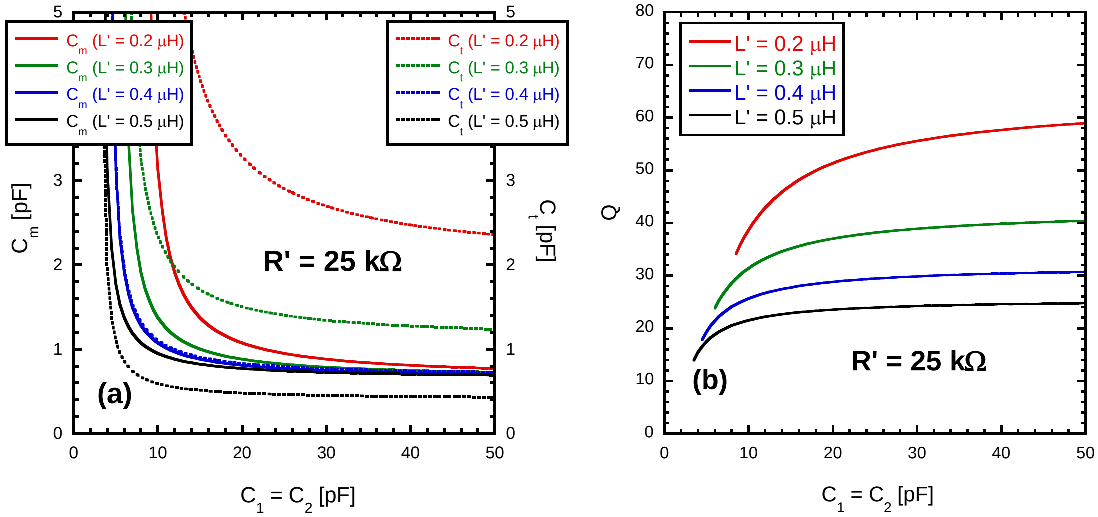

Figure S6: Consider the case $C_1 = C_2$: a) Plot of the calculated optimal tuning capacitance, C_t, and matching capacitance, C_m, of the battery-as-coil circuit as a function of the two capacitances, C_1 and C_2, for a range of inductances, L', and an effective parallel resistance, R', of 25 kΩ; b) Plot of the loaded Q of the circuit as a function of the two capacitances, C_1 and C_2. The loaded Q was found from direct calculations of the impedance as a function of frequency

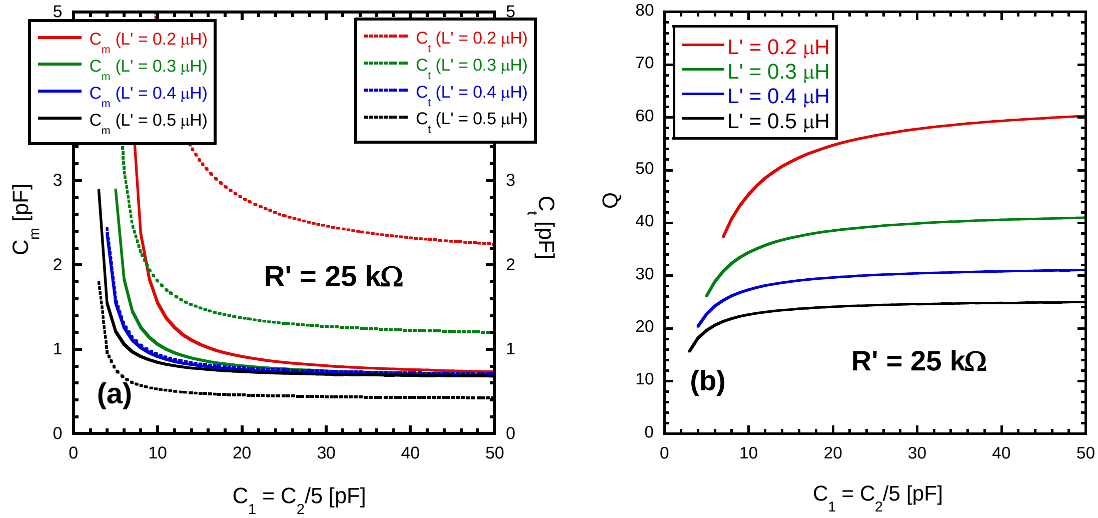

Figure S7: Consider the case $C_1 = C_2/5$: a) Plot of the calculated optimal tuning capacitance, C_t, and matching capacitance, C_m, of the battery-as-coil circuit as a function of the two capacitances, C_1 and C_2, for a range of inductances, L', and an effective parallel resistance, R', of 50 kΩ; b) Plot of the loaded Q of the circuit as a function of the two capacitances, C_1 and C_2. The loaded Q was found from direct calculations of the impedance as a function of frequency

L (μH)	R (kΩ)	C_m (pF)	C_t (pF)	Q (loaded)
0.4	100	0.46	2.18	128.1
0.4	50	0.65	1.99	63.8
1.0	100	0.46	0.60	51.2
1.0	50	0.65	0.41	25.6

Table S1: Results from impedance calculations for the optimal tuning at 155 MHz for the circuit shown in Figure 2a as a function of the relevant circuit elements.

L' (μH)	R' (kΩ)	C_1 (pF)	C_2 (pF)	C_m (pF)	C_t (pF)	Q (loaded)
0.2	50	10	10	2.21	10.41	77.4
0.2	50	20	20	0.76	3.60	102.7
0.2	*50*	*5000*	*5000*	*0.46*	*2.18*	*127.9*
0.2	25	10	10	3.14	9.52	38.6
0.2	25	20	20	1.08	3.29	51.3
0.2	*25*	*5000*	*5000*	*0.65*	*1.99*	*63.9*
0.5	50	10	10	0.67	0.87	43.1
0.5	50	20	20	0.55	0.71	47.1
0.5	*50*	*5000*	*5000*	*0.46*	*0.60*	*51.2*
0.5	25	10	10	0.95	0.59	21.5
0.5	25	20	20	0.77	0.48	23.5
0.5	*25*	*5000*	*5000*	*0.65*	*0.41*	*25.6*

Table S2: Results from impedance calculations of the optimal tuning matching and tuning capacitances, C_m and C_t, at 155 MHz for the resonant circuit of Figure 2b as a function of the values of the other components in these circuits. Italic values mark value pairs, which make circuits in Figure 2a and Figure 2b equivalent.

References

1. Miller, J.B., et al., *Interplay among recovery time, signal, and noise: Series- and parallel-tuned circuits are not always the same.* Concepts in Magnetic Resonance, 2000. **12**(3): p. 125-136.
